# Deep analysis of immune response and metabolic signature in children with food protein induced enterocolitis to cow’s milk

**DOI:** 10.1186/s13601-018-0224-9

**Published:** 2018-09-28

**Authors:** Karine Adel-Patient, Guillaume Lezmi, Florence Anne Castelli, Sibylle Blanc, Hervé Bernard, Pascale Soulaines, Pascale Dumond, Sandrine Ah-Leung, Florence Lageix, Delphine de Boissieu, Naima Cortes-Perez, Stéphane Hazebrouck, François Fenaille, Christophe Junot, Christophe Dupont

**Affiliations:** 1 Service de Pharmacologie et Immunoanalyse (SPI), CEA, INRA, Université Paris-Saclay, 91191 Gif-sur-Yvette, France; 2 APHP, Hôpital Universitaire Necker-Enfants Malades, Service de Pneumologie et Allergologie Pédiatriques, Université Paris Descartes, 75015 Paris, France; 3 Pediatric Allergology Service, Hôpital d’Enfants, 54511 Vandoeuvre les Nancy, France; 4 Nice Pediatric Hospital, CHU Lenval, 06200 Nice, France

**Keywords:** Food allergy, Non-IgE, FPIES, Cow’s milk, Mechanisms, Humoral immunity, Cellular immunity, Metabolomics

## Abstract

**Background:**

Food Protein-Induced Enterocolitis Syndrome (FPIES) is considered to be a non-IgE mediated food allergy. However, its pathogenesis remains poorly understood and biomarkers are lacking. We aimed to perform in-depth characterization of humoral and cellular immune responses in children with cow’s milk (CM)-FPIES and investigated whether there is a FPIES metabolomic signature.

**Methods:**

Children with CM-FPIES and control subjects with an IgE-mediated CM allergy (IgE-CMA), both avoiding CM, were recruited on the day of an oral food challenge. Blood samples were collected before the challenge. Total and specific levels of IgE, IgG1-4, IgA, IgM and IgD to various whey and casein allergens and to their gastroduodenal digestion products were measured in plasma, using plasma from CM-tolerant peanut allergic patients (IgE-PA, not avoiding CM) as additional controls. Cytokine secretion and cellular proliferation were analyzed after stimulation of PBMC with different CM allergens. Metabolomic profiles were obtained for plasma samples using liquid chromatography coupled to high-resolution mass spectrometry.

**Results:**

Nine children with CM-FPIES and 12 control subjects (6 IgE-CMA and 6 IgE-PA) were included. In children with CM-FPIES, total Ig concentrations were lower than in control subjects, specific Ig against CM components were weak to undetectable, and no specific IgE against CM digestion products were detected. Moreover, in CM-FPIES patients, we did not find any Th cell proliferation or associated cytokine secretion after allergen reactivation, whereas such responses were clearly found in children with IgE-CMA. Plasma metabolic profiles were different between CM allergic patients, with significantly lower concentrations of various fatty acids and higher concentrations of primary metabolites such as amino acids in CM-FPIES compared to IgE-CMA patients.

**Conclusions:**

In CM-FPIES, both humoral and cellular specific immune responses are weak or absent, and this is not related to CM avoidance. A metabolomic signature was identified in patients with CM-FPIES that may be useful for the diagnosis and management of this disease.

**Electronic supplementary material:**

The online version of this article (10.1186/s13601-018-0224-9) contains supplementary material, which is available to authorized users.

## Background

Food Protein-Induced Enterocolitis Syndrome (FPIES) is a food allergy affecting predominantly infants, with cow’s milk (CM) being the most common causative food. Symptoms depend on frequency of food exposure [1, 2]. Chronic exposure to CM results in chronic symptoms including diarrhoea and failure to thrive (FTT). Symptoms improve after CM exclusion and re-exposure leads to a typical acute digestive form including repetitive protracted vomiting starting 1–4 h after CM ingestion, often accompanied by pallor and lethargy [3]. The acute form of FPIES differs from immediate IgE-dependent allergy, notably because there is no involvement of the skin or respiratory tract. In addition, skin prick testing and specific IgE levels are usually negative, although sensitization to the triggering food has been reported in some patients (atypical FPIES) and specific IgE to other foods may be present [1, 3–5]. Resolution of FPIES occurs at different ages depending on the food and the geographical regions considered, with children presenting IgE sensitization at higher risk of a protracted course [4–7].

The pathogenesis of FPIES remains poorly described and no biomarkers are available [1]. Although a Th2-biased immunopathological response has been suggested, evidence of specific T cell activation is still lacking [1, 2, 8]. Recent data suggest a role of systemic innate cells in mediating clinical reactivity [9]. The paucity of IgG, IgG4 and IgA humoral response against casein was evidenced in patients with active FPIES vs resolved FPIES [10]. However, these analyses were based on responses to CM and casein only, whereas other CM components were not studied, and other IgG subtypes or isotypes such as IgM or IgD were not analyzed. Moreover, the reactivity of neo-epitopes produced during gastroduodenal digestion was not assessed.

A further in-depth characterization of circulating cells and antibodies in patients with FPIES would improve our understanding of the pathogenesis of FPIES and could be useful for the development of new diagnostic tools. Moreover, processes such as glycolysis, fatty acid and mitochondrial metabolism are now recognized as crucial players in immune responses [11, 12]. Pathophysiological changes associated with FPIES could then translate into a global modification of the plasma metabolome, providing a specific metabolomic signature that may help identify specific biomarkers. We investigated in depth the humoral and cellular immune response in FPIES and performed a non-targeted metabolomic analysis on plasma. We hypothesized that humoral and cellular responses are weak in FPIES and that FPIES is characterized by a specific metabolomic signature.

## Methods

### Subjects

In this descriptive study, children with a CM-FPIES were compared with age-matched control subjects. Children aged up to 10 years previously diagnosed with CM-FPIES [1, 2] were recruited in our day care unit on the day of an open food challenge (OFC) with CM performed to evaluate natural resolution of CM-FPIES. All children with CM-FPIES had a history of chronic symptoms including diarrhoea, low weight gain and/or emesis that resolved within days on CM avoidance. All of them experienced at least one typical acute FPIES episode including recurrent emesis with pallor, lethargy and dehydration with or without hypotension, occurring 1–4 h after CM ingestion, and requiring intravenous fluid perfusion in an emergency department. Exclusion criteria were a history of immediate (< 2 h) reaction, reactions with skin or respiratory tract involvement, positive SPT to CM or CM specific IgE level > 0.1 KuA/L. Children with IgE-mediated CM allergy (IgE-CMA) were included as control subjects on the day of an OFC with baked milk, performed to evaluate baked milk tolerance. Children with IgE-CMA had a clear history of immediate reaction (< 1 h) occurring after CM ingestion, with CM specific IgE levels > 0.10 kuA/L and positive skin prick test to CM (wheal > 3 mm). In those patients, OFC was not mandatory to confirm CMA [13]. All of them had recent immediate reaction following ingestion of raw CM, within the 3 months before the OFC to baked milk was performed.

Blood samples were taken before the OFC. Three other patients initially recruited as IgE-CMA children finally show no more clinical history of CM allergy, with no immediate reaction noticed after ingestion of CM, and where then considered as become tolerant (IgE-resolved). Additional plasma samples from children with an IgE-mediated peanut allergy were used as controls from allergic subjects tolerating CM.

### Reagents

$\upbeta$-Lactoglobulin (BLG), whole casein (cas) and its 4 constituents ($\upalpha$s1-cas, $\upalpha$s2-cas, $\upbeta$-cas, $\upkappa$-cas), $\upalpha$-lactalbumin ($\upalpha$-lact) and lactoferrin (LF) were purified from raw CM and characterized as previously described [14–17]. Commercial infant formulas Pregestimil (extensive hydrolysate of caseins, Mead Johnson, Nijmegen, Netherlands), Pepti-junior (extensive hydrolysate of whey proteins, Laboratoires Picot, Laval, France) and Neocate (amino acid formula, SHS International, Liverpool, UK) were locally purchased.

### Blood collection, PBMC and plasma separation

Blood samples were collected in BD Vacutainer^®^ sodium heparin tubes (BD, Le Pont de Claix, France), kept at room temperature and processed within 3–4 h. Peripheral Blood Mononuclear Cells (PBMC) were obtained from peripheral blood diluted 1:2 in AIM V^®^ Serum Free Medium (Thermo Fisher Scientific, Waltham, USA) by using Histopaque^®^-1077 (Sigma Aldrich, St Louis, USA) following the provider’s recommendations. After centrifugation (400×*g*, 30 min, + 20 °C), plasma was collected, aliquoted and kept at − 20 °C for antibody and metabolomics analysis. PBMC were collected, washed several times with PBS-EDTA 2 mM and finally suspended in RPMI-1640 medium supplemented with 5% autologous plasma, 2 mM l-glutamine, 100 U penicillin, 100 µg/mL streptomycin (All from GIBCO^®^, Thermo Fisher Scientific, Waltham, USA) for cellular analysis.

### Analysis of humoral response

#### Total Ig

Concentrations of total IgE, IgG1, IgG2, IgG3, IgG4, IgM and IgA were determined using BioPlex Pro™ Human Isotyping Panel assays and BioPlex200 apparatus from Bio-Rad (Marnes-la-Coquette, France), following the provider’s recommendations.

#### Specific Ig

Specific antibodies against CM allergens were analyzed by using the direct Enzyme AllergoSorbant Test (EAST) as partially described in [17, 18]. Assays were performed on allergen-coated 96-well microtitre plates (Immunoplate Maxisorb^®^, Nunc, Roskilde, Denmark), using AutoPlate Washer and Microfill dispenser equipment from BioTek instruments (Avantec, Rungis, France). Pre-selected and labelled (biotinylated or acetylcholinesterase-labelled) anti-human IgE (clone BS17, [19]), or anti-human IgG1 (clone JDC1), IgG2 (clone HP6002), IgG3 (clone HP6050), IgG4 (clone HP6023), IgD (clone IADB6) and IgM (clone UHB) (all from Southern Biotech, Birmingham, AL, USA), or anti-human IgA (AffiniPure F(ab’)_2_ Fragment, Jackson ImmunoResearch Laboratories, Wets Grove, USA) were used for staining. Preliminary experiments allowed selection of these antibodies, based on their specificity and sensitivity using purified standard isotypes. Biotinylation was performed in 20 mM borate buffer using an antibody:biotin molar ratio of 40 (EZ-link^®^ Sulfo-NHS-LC-LC-biotin, Thermo Scientific). Labelling of streptavidin or direct labelling of antibodies with acetylcholinesterase (AChE) was performed as previously described [19, 20]. Solid phase-bound AChE activity was determined by addition of 200 µL/well of Ellman’s reagent as an enzyme–substrate and the absorbance was measured at 414 nm using automatic reader plates (MultiskanEx, Thermo Electron Corporation, Vantaa, Finland). Plasma was tested at 3 dilutions, from 1/2 to 1/200 depending on the isotype. A signal greater than the mean of nonspecific binding + 3σ (obtained with buffer instead of plasma) was considered positive. Standard curves obtained with anti-human IgE [19] or anti-human IgG (F(ab′)2 Fragment specific, Pierce^®^, Thermo Scientific, Rockford, USA) coated plates and standard human IgE (World Health Organization; concentrations ranging from 10 to 0.08 IU/mL) or commercial isotype standard IgGs (all from AbD-Serotec, Bio-Rad, concentrations ranging from 1 µg/mL to 50 ng/mL) were used as reference to quantify antibody concentrations. Specific IgE against CM purified proteins were also assayed using reverse EAST, a more sensitive method where IgE were first captured on anti-human IgE coated plates and labelled CM-allergens were used for staining [21].

Additionally, specific IgE and IgG4 were analyzed against hydrolyzed proteins. Raw milk was digested under “physiological conditions” using pepsin and then trypsin/chymotrypsin, as previously described [22, 23]. Pepsin (from porcine gastric mucosa, 3440 U/mg of protein calculated using haemoglobin as substrate; Sigma-Aldrich) was used at an enzyme-to-substrate ratio of 172 U/mg, and trypsin (from bovine pancreas, 11,886 U/mg of protein calculated using BAEE as substrate, Sigma-Aldrich) and chymotrypsin ($\upalpha$-type VII from bovine pancreas, 52 U/mg of protein calculated using BTEE as substrate, Sigma Aldrich) were added at an enzyme-to-substrate ratio of 34.5 U/mg and 0.4 U/mg, respectively. Hydrolysates were passively immobilized on microtitre plates and specific IgE and IgG4 stained as for specific Ig.

#### IgE immunoblot

SDS-PAGE and IgE immunoblot analyses of defatted CM were performed under reducing conditions using reagents and recommendations from the provider (Invitrogen, Life Technologies, Carlsbad, USA). Defatted CM and molecular weight markers (Novex^®^ Sharp prestained protein standard) were loaded on NuPage Novex Bis–Tris Gels. Electrophoresis was performed using XCell SureLock Mini-Cell with a constant voltage of 200 V for 40 min. After electrophoresis, gels were stained with GelCode Blue Stain Reagent (Pierce, Thermo Scientific) or proteins were transferred to PVDF membranes (Hybond-P, GE-Healthcare Life Sciences) for 90 min at 25 V using a XCell II blot module. Membranes were saturated for 1 h at 20 °C with TBST (20 mM Tris, pH 7.6, 0.25 M NaCl, 0.5% Tween) supplemented with 5% BSA (Sigma-Aldrich). Plasma diluted 1:4 were incubated with slight shaking for 18 h at 4 °C. After several washings with TBST, secondary antibody (goat anti-human IgE peroxidase conjugated STAR147P, AbDSerotec-Bio-Rad) was incubated with slight shaking for 2 h at 20 °C. Membranes were then revealed with ECL Prime Western blotting detection reagent (GE-Healthcare Life Sciences) for 5 min and then analyzed using ChemiDoc™Touch Imaging System from Bio-Rad.

### Analysis of cellular immune response

#### In vitro reactivation of PBMC

After isolation of PBMC, cell count was performed using TC-10 apparatus (Bio-Rad, Marnes-la-Coquette, France). Cellular concentration was adjusted to 1 × 10^6^ cells/mL and 225 µL/well was added to 96-well culture plates. Purified proteins (final concentrations 10 and 50 µg/mL) or infant formula (final protein concentrations 500 and 100 µg/mL) was then added, and cell cultures were incubated for 6 days at 37 °C in a humidified 5% CO_2_ atmosphere. LPS content in purified proteins and formulas was checked using the Pierce™ LAL chromogenic endotoxin quantification kit (Thermo Fisher Scientific), following the provider’s recommendations. LPS contents in BLG, $\upalpha$s1-cas, $\upbeta$-cas, $\upkappa$-cas and LF were below 30 pg/mg of protein. LPS contents in whole caseins and $\upalpha$-lact was 70 pg/mg of protein, and 125 pg/mg for $\upalpha$s2-cas. Endotoxin levels were then considered as acceptable taking into account the amount of protein added during in vitro reactivation and the amount of endotoxin that will not induce non-specific activation of PBMC [24]. Pregestimil, Pepti-junior and Neocate solutions contained respectively 135, 215 and 205 pg of endotoxin/mg of protein. Medium alone (PBS) was used as a negative control, whereas phytohaemagglutinin (PHA-L, lectin from *Phaseolus vulgaris*, Sigma Aldrich, St Louis, USA) or bacterial lipopolysaccharide (LPS from *E. coli* serotype 0127:B3, Sigma Aldrich) was used as positive control (1–10 µg/mL). In some experiments, cells were stained with 1 µM CFSE (CFSE Cell Division Tracker kit, Biolegend, San Diego, USA) following the provider’s recommendation before reactivation.

#### Cytokine production analysis

After in vitro reactivation, plates were centrifuged and supernatants collected, aliquoted and kept at − 80 °C until further assay. Cytokines (IL-1$\upbeta$, IL-2, IL-3, IL-5, IL-6, IL-10, IL-13, IL-17A, IFN$\upgamma$, TNF-$\upalpha$) were assayed using BioPlex Pro™ Human cytokine kits and BioPlex200^®^ apparatus, following the provider’s recommendations (Bio-Rad, Marnes-la-Coquette, France).

#### Flow cytometry analysis

In some patients, cultured cells obtained after in vitro reactivation were suspended in PBS Ca^−^/Mg^−^, 2 mM EDTA, 5% heat-inactivated foetal calf serum (FCS) and extracellular and intracellular labelling of Th and Treg cells was performed using optimized antibody panels. For Th cells, analysis was performed as described in [25], using anti-human CD4 (clone OKT4, Brilliant Violet 785™), CD25 (clone M-A053, PE/Dazzle™ 594), CD45RA (Clone HI100, Brilliant Violet 510™), CCR6 (CD196, clone G034E3, PE-Cy7), CXCR3 (CD183, clone G025H7, Brilliant Violet 421™) and CCR4 (CD194, clone L291H4, Brilliant Violet 605™); for Treg analysis, we used anti-human CD3 (clone UCHT1, Brilliant Violet 605™), CD25 (clone M-A251, Brilliant Violet 421™), CD4 (clone RPA-T4, Brilliant Violet 785™), Helios (clone 22F6, PE) and Foxp3 (clone 206D, Alexa Fluor^®^ 647). All antibodies were from Biolegend (San Diego, USA). Intracellular labelling of Foxp3 and Helios was performed after fixation/permeabilization using the Foxp3 staining kit from Miltenyi Biotec (Miltenyi Biotec GmbH, Bergisch Gladbach, Germany) following the provider’s recommendations.

Blood and biopsies of the jejunum, sigmoid and rectum were obtained from two brothers suffering from FPIES, and not initially included in our cohort: one resolved under a strict elimination diet (Neocate; age 38 months), the other one had an active FPIES with typical acute symptoms few days before the endoscopy, after ingestion of wheat and corn. Plasma and PBMC were isolated as above. Both demonstrated plasma humoral responses in line with that observed in our CM-FPIES population (not shown). Biopsy material was immediately placed in tissue storage solution (Miltenyi Biotec GmbH) and processed within 24 h. Biopsy material was washed in RPMI-1640 medium and tissue was digested using Liberase™ (Research Grade, 1 mg/mL, Roche Diagnostics GmbH, Sigma-Aldrich) and DNase I (0.02 mg/mL, Invitrogen, Life Technologies) for 45 min at 37 °C followed by mechanical dissociation using gentleMACS^®^ C tubes and the gentleMACS™ Dissociator (Miltenyi Biotec GmbH). After washing, cells were suspended in PBS, 5% FCS, 2 mM EDTA and staining for flow cytometry was performed as above and using the following reagents and antibodies for viability assessment and extracellular or intracellular labelling: Fixable Yellow Dead Cell Stain Kit (Thermo Fisher Scientific), lineage (lin; anti-human CD3, CD11c, CD14, CD16, CD19, CD56 (NCAM), Fc$\upvarepsilon$RI$\upalpha$, CD1a, CD123; APC-Vio770™), and anti-human CD127 (IL-7R$\upalpha$, PE-Vio615™), CD4 (VioGreen^®^), CD45 (PerCP-Vio700™), CD294 (CRTH2; PE-Vio770™), T-bet (PE), ROR$\upgamma$t (APC), GATA3 (FITC) all from Miltenyi Biotec and anti-human-IL-13 (BV711, BD Biosciences, Le Pont de Claix, France), anti-human IL-22 (eFluor 450, Affymetrix eBiosciences, Thermo Fisher Scientific), anti-human-IFN$\upgamma$ (BD Bioscience).

Approximately 70,000 cells were collected using a NovoCyte flow cytometer (ACEA Bioscience, Inc.) and analysis was performed using NovoExpress™ Software (Version 1.2.1, ACEA Biosciences, Inc.). Samples were first inspected in all light scatter patterns and fluorescence channels to confirm quality and abnormal cells (dead cells, aggregates…) were excluded. Each acquisition contained unlabelled samples, single-stained cells and/or an FMO strategy for reporting percentage of positive cells and compensation.

### Statistical analysis for humoral and cellular analysis

Non-parametric tests were performed using the Mann–Whitney *t* test (comparison between specified groups), the Kruskal–Wallis test and Dunn’s multiple comparison post-test to compare all the groups together, or the Wilcoxon sign rank test (comparison of different treatments/Assays for patients in the same group). A *p* value < 0.05 was considered to be significant. All statistical analyses were performed using GraphPad Prism version 5.01 for Windows (GraphPad Software, San Diego, CA, USA).

### Metabolomic analysis

#### Metabolite extraction

Metabolites were extracted from 50 µL of plasma as previously described [26]. Briefly, for each sample, 2 aliquots of 50 μL of plasma were treated with 200 μL of methanol, vortexed for 20 s and left on ice for 30 min to allow protein precipitation. Samples were then centrifuged for 20 min at 15,000×*g*. Supernatants were collected and dried under nitrogen. Dried extracts were dissolved in 150 µL of H_2_O/ACN (95/5%) for C18 analysis or ammonium carbonate 10 mM pH 10.5/ACN (40/60%) for HILIC analysis.

#### Metabolite detection: instrumentation and LC/MS acquisitions

Extracts were analyzed by liquid chromatography (LC) coupled to mass spectrometry, as previously described [26] using a Dionex Ultimate chromatographic system coupled to an Exactive (Orbitrap) mass spectrometer from Thermo Fisher Scientific (Courtaboeuf, France) fitted with an electrospray source operated in the positive and negative ion modes. The software interface was Xcalibur (version 2.1) (Thermo Fisher Scientific, Courtaboeuf, France).

Ultra high-performance LC (UHPLC) separation was performed on a Hypersil GOLD C18 (1.9 μm, 2.1 mm × 150 mm) column at 30 °C (Thermo Fisher Scientific, les Ulis, France). Mobile phases for reverse phase columns were 100% water in A and 100% ACN in B, both containing 0.1% formic acid. Chromatographic elution was achieved with a flow rate of 500 μL/min. After injection of 10 μL of sample, elution consisted of an isocratic step of 2 min at 5% phase B, followed by a linear gradient from 5 to 100% of phase B for the next 11 min. These proportions were kept constant for 12.5 min before returning to 5% B for 4.5 min. The column effluent was directly introduced into the electrospray source of the mass spectrometer and analyses were performed in the positive ion mode. Source parameters were as follows: droplet evaporation temperature 280 °C; capillary voltage, 5 kV; sheath gas pressure and the auxiliary gas pressure, respectively at 60 and 10 arbitrary units with nitrogen gas; mass resolution power, 50,000 m/Δm; full width at half maximum (FWHM) at m/z 200, for singly charged ions; detection from m/z 85 to 1000.

The high-performance LC (HPLC) separation was performed on a Sequant ZICpHILIC column (5 μm, 2.1 × 150 mm) at 15 °C (Merck, Darmstadt, Germany). Mobile phase A consisted of an aqueous buffer of 10 mM ammonium carbonate pH 10.5, and mobile phase B of 100% ACN. Chromatographic elution was achieved with a flow rate of 200 μL/min. After injection of 10 μL of sample, elution started with an isocratic step of 2 min at 80% B, followed by a linear gradient from 80 to 40% of phase B from 2 to 12 min. The column effluent was directly introduced into the electrospray source of the mass spectrometer, and analyses were performed in the negative ion mode. Source parameters were as follows: droplet evaporation temperature, 280 °C; capillary voltage, − 3 kV; sheath gas pressure and the auxiliary gas pressure, respectively at 60 and 10 arbitrary units with nitrogen gas; mass resolution power, 50,000 m/Δm; full width at half maximum (FWHM) at m/z 200, for singly charged ions; detection from m/z 85 to 1000.

#### Data processing and statistical analysis

Data processing workflow and statistical analyses were performed on the open web-based platform workflow4metabolomics (W4M: http://workflow4metabolomics.org), a collaborative research infrastructure for computational metabolomics [27]. Automatic peak detection and integration were performed using the matched filter algorithm in the W4M pre-processing package (including XCMS software). All raw data were manually inspected using the Qualbrowser module of Xcalibur, while the Quanbrowser module was used for peak detection and integration of internal standards. To remove analytical drift induced by clogging of the ESI source observed in the course of analytical runs, chromatographic peak areas of each variable present in the XCMS peak lists were normalized using the LOESS algorithm (W4M package). Features generated from XCMS were filtered according to the following criteria: (i) the correlation between QC dilution factors and areas of chromatographic peaks (filtered variables should have coefficients of correlation above 0.7 to account for metabolites occurring at low concentrations and which are no longer detected in the most diluted samples) (ii) repeatability (the coefficient of variation obtained on chromatographic peak areas of QC samples should be below 30%), and (iii) ratio of chromatographic peak areas of biological to blank samples above a value of 3.

Statistical analyses were performed with W4M (multivariate and univariate statistical tests), Simca P (multivariate PLS-DA models) or Prism (univariate tests) software tools. The discriminant metabolites were selected by combining multivariate variable importance in the projection (VIP) obtained from the PLS-DA model and univariate *p* values (nonparametric Mann–Whitney statistical test). The metabolites were considered as discriminant when VIP > 1.5 and *p* value < 0.1.

#### Metabolite annotation and LC/ESI–MS–MS validation

Feature annotation was performed considering a ± 10 ppm mass tolerance and using our in-house spectral database [26, 28], as well as the publicly available databases KEGG [29], HMDB [30] and METLIN [31]. To be identified, ions had to match at least two orthogonal criteria among accurate measured mass, isotopic pattern, MS/MS spectrum, and retention time; and to those of an authentic chemical standard analyzed under the same analytical conditions, as proposed by the Metabolomics Standards Initiative [32]: level 1 (identified): based on accurate mass, column retention time similarity with a standard and MS/MS spectrum. Level 3 (putatively characterized): based on accurate mass, and interpretation of MS/MS spectra. Metabolite identification was further confirmed by additional LC/ESI–MS–MS experiments, performed using a Dionex Ultimate chromatographic system combined with a Q-Exactive Plus mass spectrometer (Hilic) or a Fusion mass spectrometer (C18), under nonresonant collision-induced dissociation conditions using higher-energy C-trap dissociation (HCD), at normalized collision energies (NCEs) 10, 20, 40 and 80%.

## Results

### Population

Nine out twelve of the CM-FPIES children recruited had a positive challenge on the day of the OFC, reacting at 45 mL of raw CM or less, with 1 having a hypovolemic shock. The six IgE-CMA diagnosed patients tolerated baked milk challenge, and were then advised to consume baked milk (but not raw milk) on a daily basis.

Nine children with CM-FPIES and six children with IgE-CMA were then included in the present study. The general characteristics of included patients are shown in Table 1. Children in both groups shared similar general characteristics (age, gender), except for IgE testing and skin prick tests that were negative in all CM-FPIES patients.

**Table 1:** Clinical characteristics of patients

	CM-FPIES	IgE-CMA	*p*
Number	9	6	
Age (years)	2.3 [1.8–2.7]	2.8 [1.9–6.9]	0.52
Gender (male/female)	4/5	5/1	0.17
Positive SPT for CM n (%)	0 (0)	6 (100)	< 0.001
Total IgE (KuA/L)	11.2 [8–25]	286.5 [78.8–444.8]	0.001
CM specific IgE (KuA/L)	0 [0–0]	22 [13.5–38.8]	< 0.001
Positive APT for CM n (%)	0 (0)	NA	

Data are expressed as the median [interquartile range] or (percentage)

*CM* Cow’s milk, *SPT* skin prick test, *APT* atopy patch test, *NA* not assessed

Plasma from CM-tolerant patients with IgE-dependent peanut allergy (IgE-PA, *n* = 6) were selected to be age-matched with the CM-allergic patients (median 2.1 years, [1.3–4.4]). All the patients had confirmed peanut allergy based on objective clinical manifestations occurring immediately after peanut ingestion, positive prick testing and specific IgE levels to peanut (not shown).

### Paucity of the humoral response in CM-FPIES patients

#### Total antibody levels are lower in FPIES patients

**Fig. 1:**
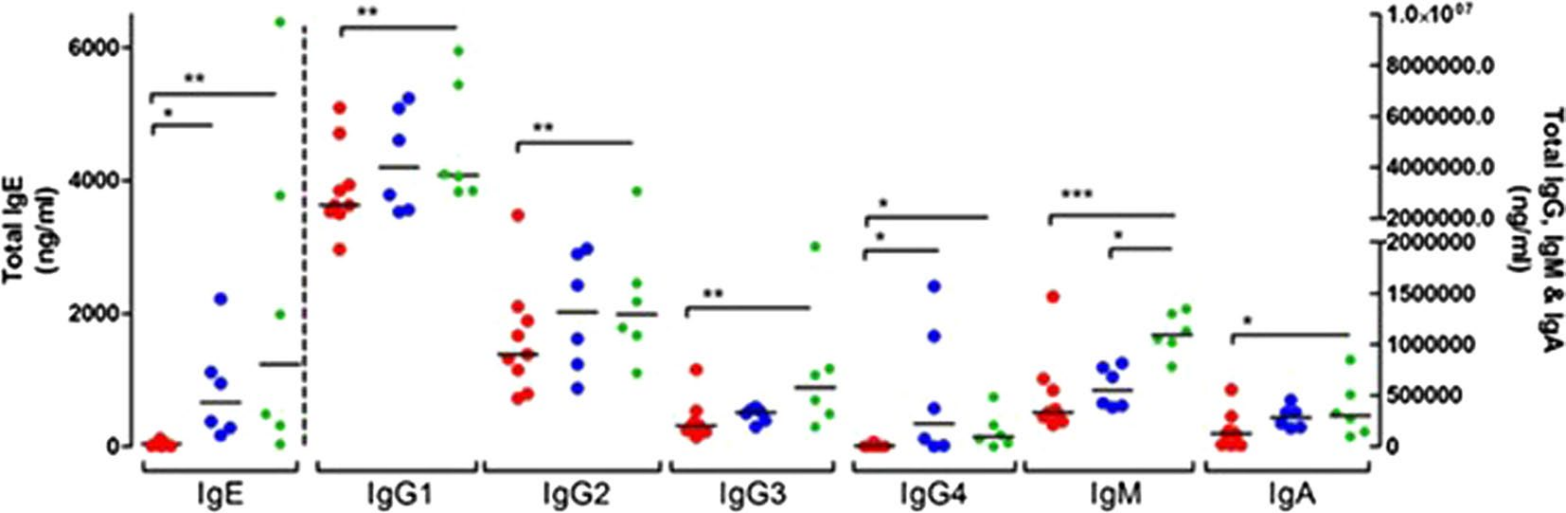
Total IgE, IgGs, IgM and IgA (ng/mL) in children with CM-FPIES (red), IgE-CMA (blue) or IgE-PNA (green). *p < 0.05, **p < 0.01, ***p < 0.001 using Kruskal–Wallis and Dunn’s multiple comparison test

Children with CM-FPIES had lower levels of total IgE and IgG4 compared with children with IgE-CMA, and they had lower levels of all total isotypes compared with those having IgE-PA (Fig. 1). Conversely, the concentrations of all isotypes except IgM were comparable in control subjects (IgE-CMA vs IgE-PA).

#### No or weak specific antibody levels were evidenced in CM-FPIES patients

##### Specific IgE

**Fig. 2:**
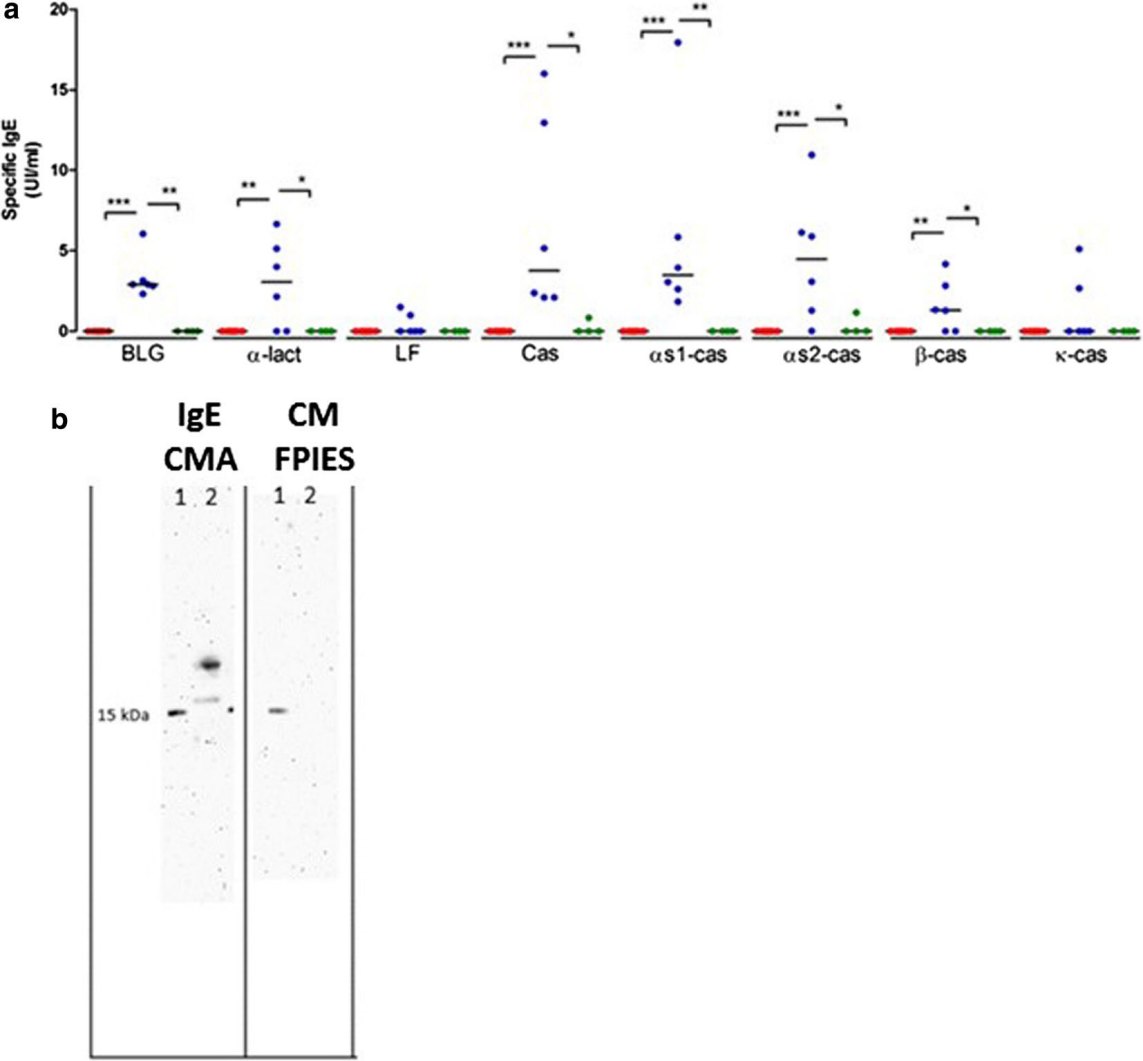
**a** Specific IgE (IU/mL) in patients with CM-FPIES (red), IgE-CMA (blue) or IgE-PA (green) assayed using immunoenzymatic assays. *p < 0.05, **p < 0.01, ***p < 0.001 using the Kruskal–Wallis test and Dunn’s multiple comparison test. **b** Representative IgE immunoblot of CM proteins using plasma from children with IgE-CMA (left) or FPIES-CMA (right). Lane 1: MW markers, Lane 2: CM proteins

Children with IgE-CMA had detectable specific IgE against BLG, $\upalpha$-lact, casein and its components $\upalpha$s-1cas, $\upalpha$s2-cas and $\upbeta$-cas (Fig. 2a). Specific IgE against LF and $\upkappa$-cas were only detected in two of the six IgE-CMA patients. Conversely, children with CM-FPIES or with IgE-PA did not have any detectable specific IgE against any of the CM allergens tested. A more sensitive immunoassay based on IgE capture confirmed these results [17, 21] (not shown). Additionally, using plasma from children with IgE-CMA, the IgE immunoblot revealed IgE binding to BLG (MW around 18 kDa) and casein (MW around 28–35 kDa), whereas no bands were observed with plasma from children with CM-FPIES (Fig. 2b).

##### Specific IgGs

**Fig. 3:**
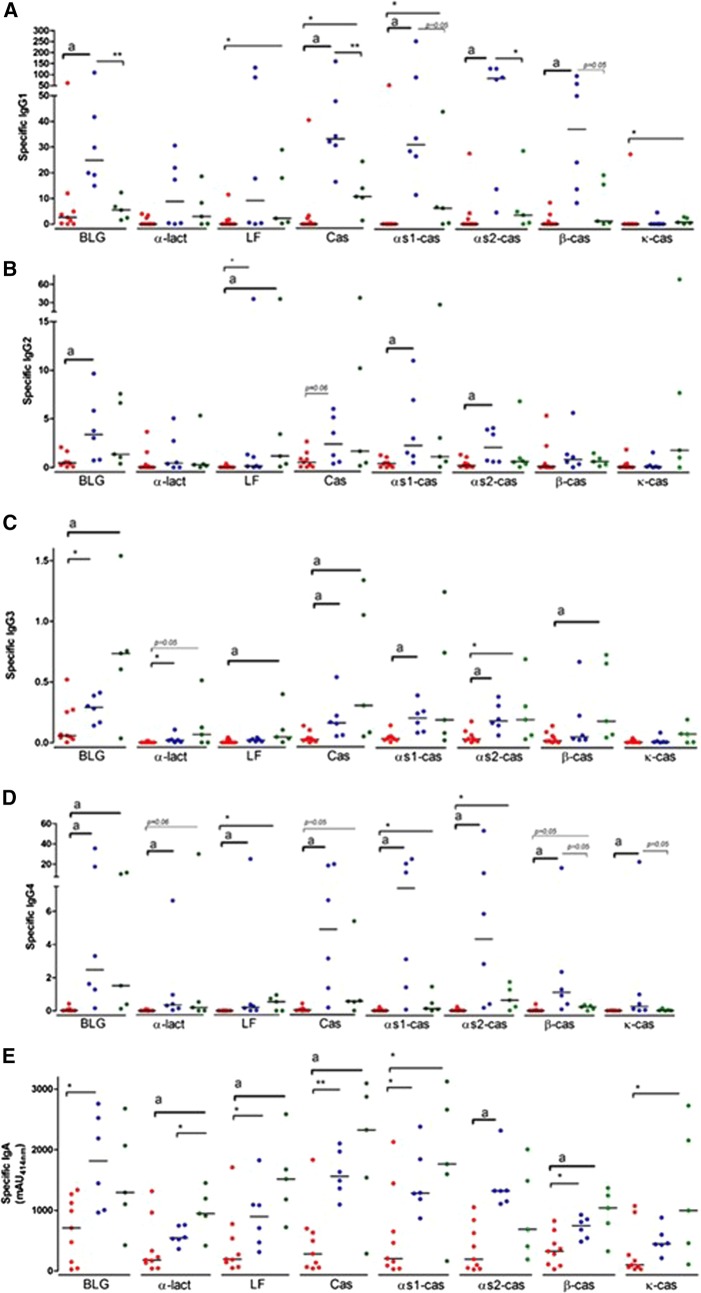
Specific IgG1 (**A**), IgG2 (**B**), IgG3 (**C**), IgG4 (**D**) and IgA (**E**) in patients suffering from CM-FPIES (red), IgE-CMA (blue) or IgE-PNA (green). Values are given as µg/mL except for IgA where values are given as milli-Absorbance Unit at 414 nm. Bars indicate medians. Statistics *p < 0.05, **p < 0.01, ***p < 0.001 using Mann–Whitney test. “a” indicates a statistical difference using the Kruskal–Wallis test and Dunn’s multiple comparison test

Overall, the concentrations of specific IgGs were weak in children with CM-FPIES, and lower than in control subjects (Fig. 3A–D). Specific IgG1 against BLG, $\upalpha$s1-cas, $\upalpha$s2-cas and β-cas were lower in children with CM-FPIES than in those with IgE-CMA. Specific IgG1 against LF, casein, $\upalpha$s1-cas and κ-cas were lower in children with CM-FPIES than in those with IgE-PA. IgG2 levels for BLG, $\upalpha$s1-cas, $\upalpha$s2-cas and β-cas were lower in children with CM-FPIES than in children with IgE-CMA, whereas specific IgG2 levels were similar between children with CM-FPIES and IgE-PA. The most striking differences were observed for specific IgG3 and IgG4. Although some values were dispersed, we found significantly lower levels of IgG3 and IgG4 against almost all CM components in children with CM-FPIES when compared with the other groups. Specific IgG4 were rather higher in children with IgE-CMA (who were avoiding CM consumption) than in those with IgE-PA (who were not).

##### Specific IgA, IgM and IgD

Specific IgA levels against BLG, LF, Cas, $\upalpha$s1-cas, $\upalpha$s2-cas and $\upbeta$-cas were significantly lower in children with CM-FPIES than in those with IgE-CMA and the specific IgA response was globally lower in CM-FPIES than in CM-tolerant children with IgE-PA (Fig. 3E). We did not detect any specific IgM or IgD in children with CM-FPIES or IgE-CMA, even using plasma diluted 1:2 (data not shown).

##### Specific IgE and IgG4 after enzymatic digestion of CM

**Fig. 4:**
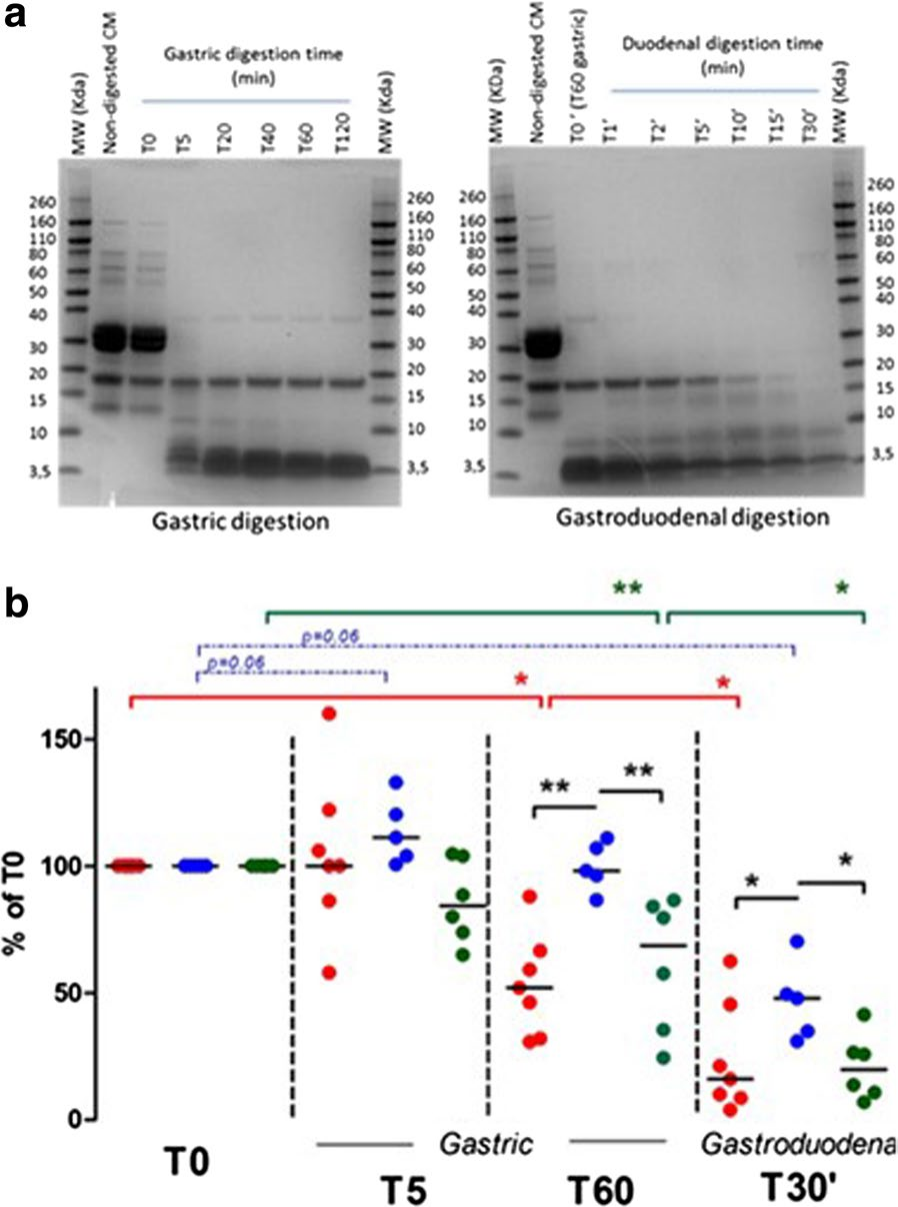
Specific IgG4 against gastroduodenal digestion products. **a** Electrophoresis of gastroduodenal CM protein digestion products: CM was digested for 0 to 120 min under physiological conditions using pepsin (T0 to T120, gastric digestion). Digestion products obtained after 60 min of pepsin digestion (T60 gastric, TO’) were then submitted to trypsin/chymotrypsin physiological digestion for 1 to 30 min (T1′ to T30′, gastroduodenal digestion). **b** Undigested CM proteins (T0), gastric digestion products obtained at 5 (T5) and 60 min (T60) and gastroduodenal digestion products obtained after 60 min of gastric digestion and 30 min of duodenal digestion (T30′) were immobilized on plates, and specific IgG4 were assayed using individual plasma (FPIES-CMA: red, IgE-CMA: blue, IgE-PNA: green). Absorbance obtained at T0 was used as an internal reference for each patient (100%). Statistics: bars and asterisks indicate statistical differences between specified time points and T0 in one group of patients (*p < 0.05, **p < 0.01; Wilcoxon sign rank test) or significant differences between groups at a given time of digestion (*p < 0.05, **p < 0.01; Mann–Whitney t test)

No specific IgE against digestion products were detected in children with CM-FPIES (not shown). Although CM proteins were already highly degraded after 5 min of gastric digestion, except for BLG (MW around 18 kDa, Fig. 4a), the binding of specific IgG4 was not significantly affected whatever the group considered (Fig. 4b, T0 vs T5). In children with CM-FPIES and IgE-PA, but not in those with IgE-CMA, the binding of specific IgG4 was significantly decreased after 60 min of gastric digestion (T60), and then further after additional duodenal digestion (T30′).

### Absence of T cell specific immune response in PBMC from children with CM-FPIES

#### Cytokine secretion

Stimulation of PBMC from children with CM-FPIES and IgE-CMA with mitogens PHA and LPS induced a significant and comparable secretion of IL-2 (as a marker of T cell activation) and of pro-inflammatory (IL-1$\upbeta$, IL-6, TNF$\upalpha$), regulatory (IL-10), Th1 (IFN$\upgamma$) and Th17 (IL-17) cytokines (see Additional file 1, “Mitogen reactivation”). A trend to lower Th2 (IL-5 and to a lesser extent IL-13) cytokine secretion was found in CM-FPIES patients.

**Fig. 5:**
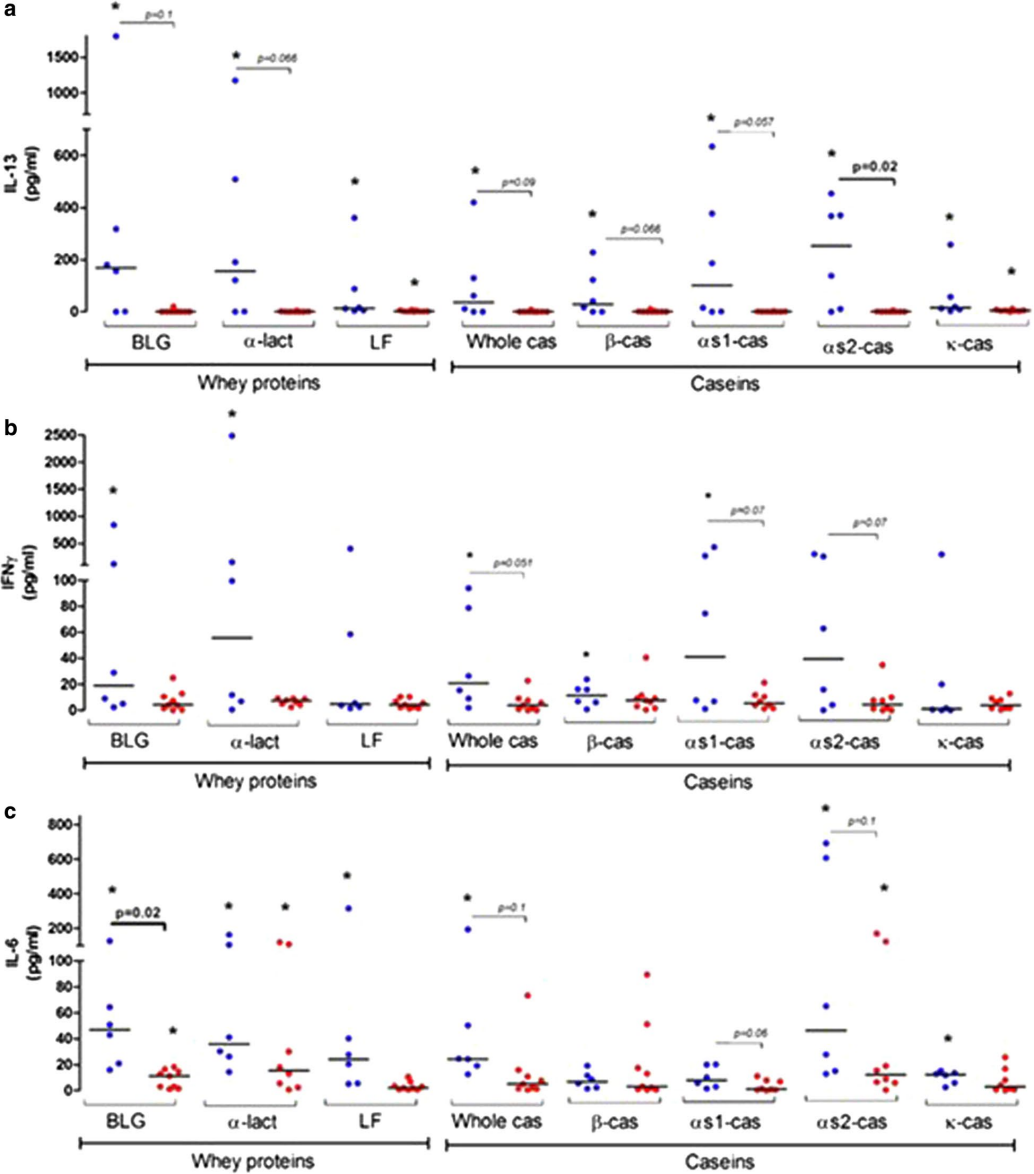
IL-13 (**a**), IFN$\upgamma$ (**b**) and IL-6 (**c**) secreted after specific reactivation of PBMC from IgE-CMA (blue) or FPIES-CMA (red) patients. PBMC were stimulated for 6 days with 50 µg/mL of purified allergens and cytokines were assayed in supernatants. Results are expressed as individual values and medians (bar) obtained for the PBMC from IgE-CMA (n = 6) or FPIES-CMA (n = 9) patients. * indicates a difference between control (PBS and/or Ara h 2) and stimulated PBMC in the same group of patients (*p* < 0.05 using Wilcoxon sign rank test); Significant difference or trends between IgE-CMA and FPIES-CMA patients are also mentioned with the associated *p* value (Mann–Whitney t test)

No cytokine secretion was detected after stimulation of PBMC with buffer alone or with purified Ara h 2 (Table 2). Stimulation with purified CM allergens and hydrolyzed infant formulas in children with CM-FPIES induced no or weak secretion of inflammatory Th2 or Th17 cytokines, whereas in children with IgE-CMA, cytokines, mainly IL-13, IL-5, IFN$\upgamma$, IL-6, and TNF$\upalpha$, were significantly induced for almost all tested allergens (Fig. 5 and Table 2).

**Table 2:**
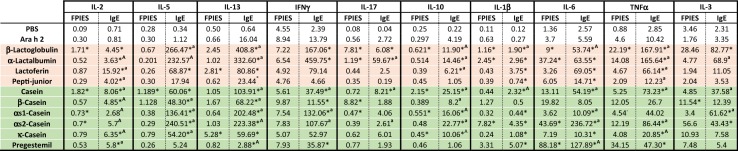
Cytokine secretion induced by buffer alone (PBS) or purified Ara h 2 as controls, or induced by purified proteins or commercial hydrolysates from whey (pink rows), or from casein fraction (green rows). Results are expressed as mean obtained for the PBMC from IgE-CMA (n = 6) or FPIES-CMA (n = 9) patients stimulated with 50 µg/mL of purified proteins or 500 µg/mL of protein hydrolysates

* Indicated a difference between control (PBS and/or Ara h 2 stimulated) and stimulated PBMC in the same group of patients (*p *< 0.05 using the Wilcoxon sign rank test); “A” indicates a significant difference between IgE-CMA and FPIES-CMA patients (*p *< 0.05, Mann–Whitney test). “a” indicates a difference trend between IgE-CMA and FPIES-CMA patients (0.05 < *p* < 0.1, Mann–Whitney test)

#### Analysis of activated T-cells by flow cytometry

After 6 days of culture, cells from some of the children with CM-FPIES (n = 6) and IgE-CMA (n = 5) were analyzed by flow cytometry. Non-stimulated cells and cells stimulated with PHA showed the same percentage of CD45RA^−^ and CD45RA^+^ cells among CD4^+^ single cells (not shown). Among CD4^+^ cells, CD25^−^CD45RA^−^ cells were selected and analyzed for Th1, Th2, Th17 and non-conventional Th1 (Th1*) memory cells [25]. Percentages of these subpopulations were comparable in non-stimulated PBMC from both groups (Additional file 2, “Memory cells”), with a higher proportion of Th1 memory cells. After non-specific stimulation with PHA, we observed a comparable percentage of Treg cells, Th1 and Th17 memory cells in PBMC from both groups, and a significantly lower percentage of Th2 memory cells in children with CM-FPIES vs those with IgE-CMA (Additional file 2, “Memory cells”).

**Fig. 6:**
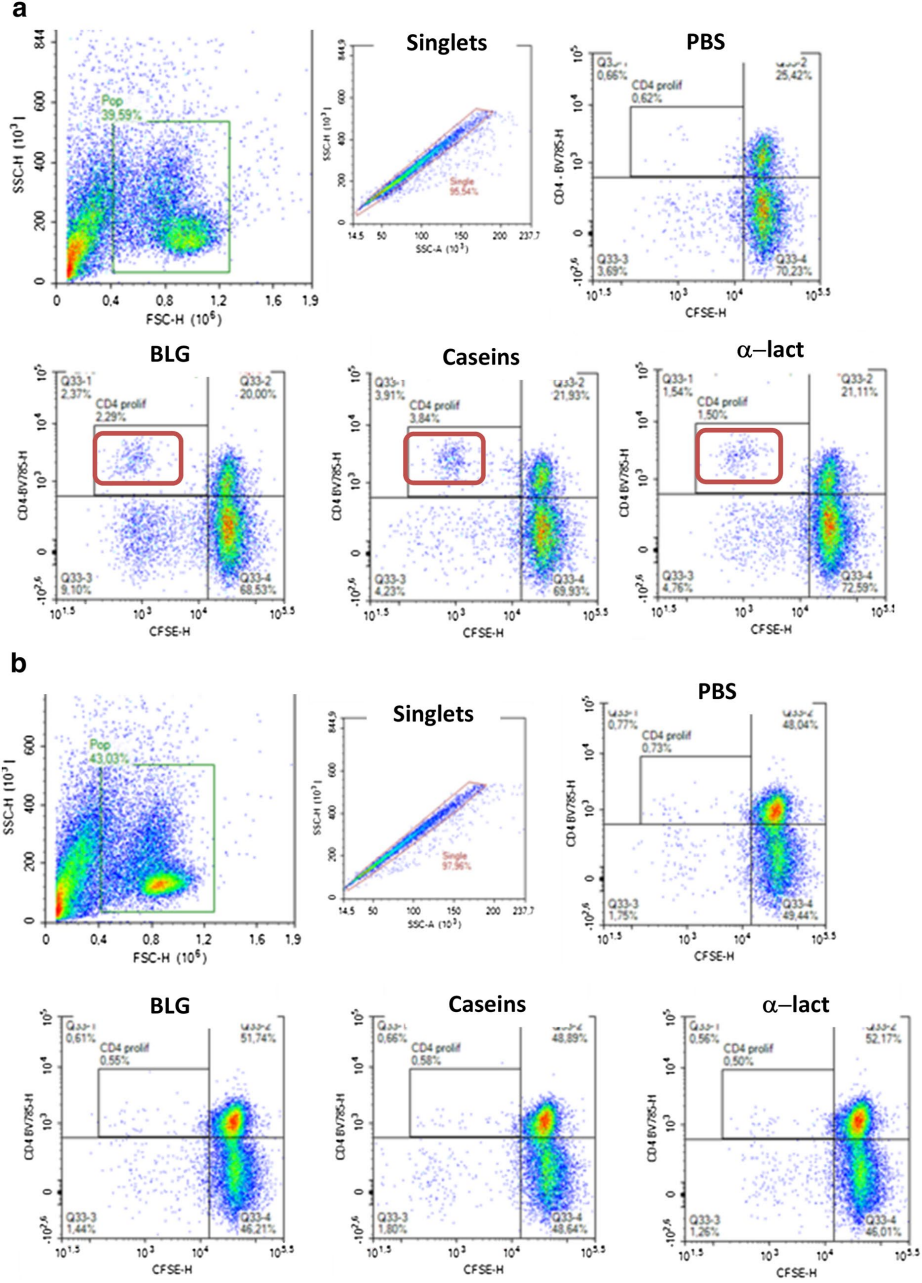
Analysis of proliferative T cells in CMA patients after allergen reactivation. PBMC from IgE-CMA (**a**) or FPIES-CMA (**b**) patients were labelled with CFSE and then cultured for 6 days with PBS or allergens purified from cow’s milk. Cells were then recovered and labelled with anti-human CD4. Among SSC-FSC gated cells, single cells were selected and analyzed for CD4 expression and CFSE signal. Proliferative T cells are defined as CD4^+^CFSE^low^ cells within a selected population (red square). Proliferative cells after reactivation with PBS, BLG, caseins or $\upalpha$-lact are shown

CFSE labelling was also performed to further analyze proliferating T cells after ex vivo stimulation. In some patients with IgE-CMA, we observed proliferating T cells after PHA (not shown) and after purified allergen stimulations (CD4^+^CFSE^−^ population, Fig. 6a). The positive patients correspond to those with the highest cytokine secretion, but the number of proliferative cells was too low to perform relevant Th memory analysis. Conversely, although PBMC from children with FPIES patients showed proliferating cells after PHA restimulation (not shown), no significant proliferation of T cells after allergen stimulation was evidenced (Fig. 6b).

### T-cells and ILC are detectable in mucosa from active FPIES children

**Fig. 7:**
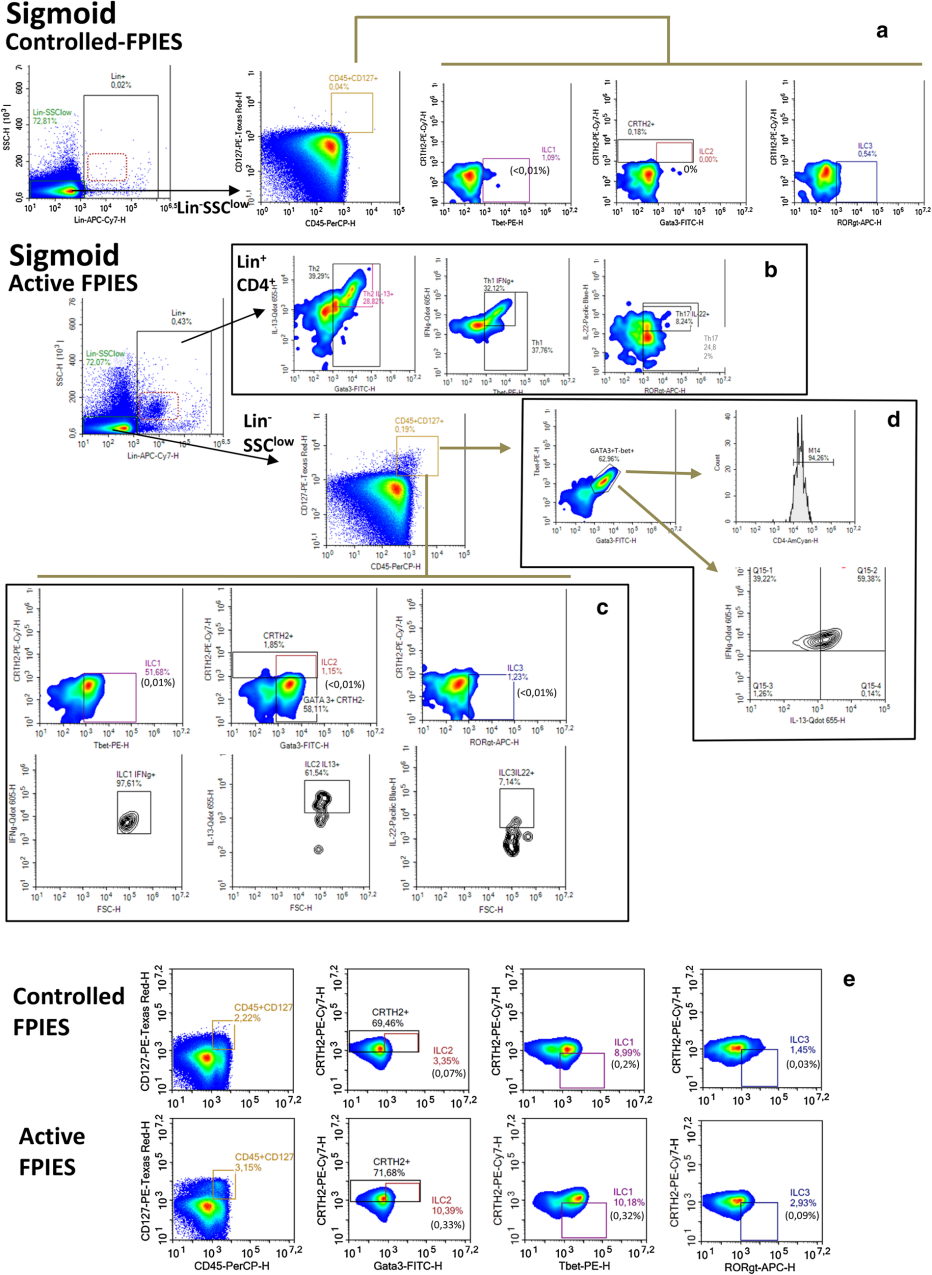
Cellular analysis in intestinal biopsies and PBMC from active versus controlled FPIES. Cellular analysis was performed on cells extracted from sigmoid biopsies obtained from children presenting controlled FPIES (non-active) or active FPIES. Cells were labelled and analyzed by flow cytometry using a NovoCyte flow cytometer and analysis was performed using NovoExpress™ software (Version 1.2.1; ACEA Biosciences, Inc.). Percentages of ILC1; ILC2, or ILC3 cells among parent cells (CD45^+^CD127^+^) and grandparent cells (Lin^−^SSC^low^) are indicated in colour and in brackets, respectively. Lin: mix of labelled anti-human CD3, CD11c, CD14, CD16, CD19, CD56, Fc$\upvarepsilon$RI$\upalpha$, CD1a, CD123

Our results question the presence of circulating specific Th cells in CM-FPIES patients and suggest the presence of other cell types and/or that induced adaptive cells are located in the mucosa and then not detectable in the periphery. Additional and very preliminary flow cytometry experiments were performed using intestinal biopsy material obtained from one patient with controlled CM-FPIES and from one patient with active FPIES. In cells extracted from rectum (not shown) and sigmoid (Fig. 7) biopsies from the patient with active FPIES, but not from those from the patient with controlled FPIES, we found SSC^med/high^Lin^+^ cells (dotted red square). These cells were mainly CD4^−^ (not shown) and may correspond to eosinophils, neutrophils and/or mast cells. Among Lin^+^ cells, very few CD4^+^ cells were found in the patient with controlled FPIES (not shown), whereas CD4^+^ cells were clearly present in the patient with active FPIES. Analysis of expression of transcription factors GATA-3, T-bet and ROR$\upgamma$t showed the presence of Th2, Th1 and Th17 cells, respectively (Fig. 7b, Lin + CD4 + gating). Some of these Th2/1/17 cells were activated, as shown by significant expression of IL-13, IFN$\upgamma$ and IL-22, respectively. This thus suggests the presence of activated T-cells in the mucosa of patients with active FPIES.

We also further analyzed the cells in the SSC^low^Lin^−^ gate. Within this population, higher percentages of CD45^+^CD127^+^ cells in the sigmoid (Fig. 7) and rectum (not shown) were evidenced in intestinal mucosa from the patient with active FPIES vs the controlled CM-FPIES patient. This resulted from a higher frequency of ILC1 (CRTH2^−^Tbet^+^), ILC2 (CRTH2^+^Gata-3^+^) and ILC3 (CRTH2^−^ROR$\upgamma$t^+^) cells in the active FPIES patient, part of which expressed associated cytokines (Fig. 7c). A very large increase in CRTH2^−^GATA3^+^ cells was also observed among CD45^+^CD127^+^ cells of active FPIES patients. These cells co-express GATA-3 and T-bet, but also IL-13 and IFN$\upgamma$ cytokines, and are CD4^+^ (Fig. 7d). Interestingly, an increased frequency of classic ILC2, and to a lesser extent of ILC1, was also detected in the PBMC of the active FPIES patient (Fig. 7e).

### Metabolomics

**Fig. 8:**
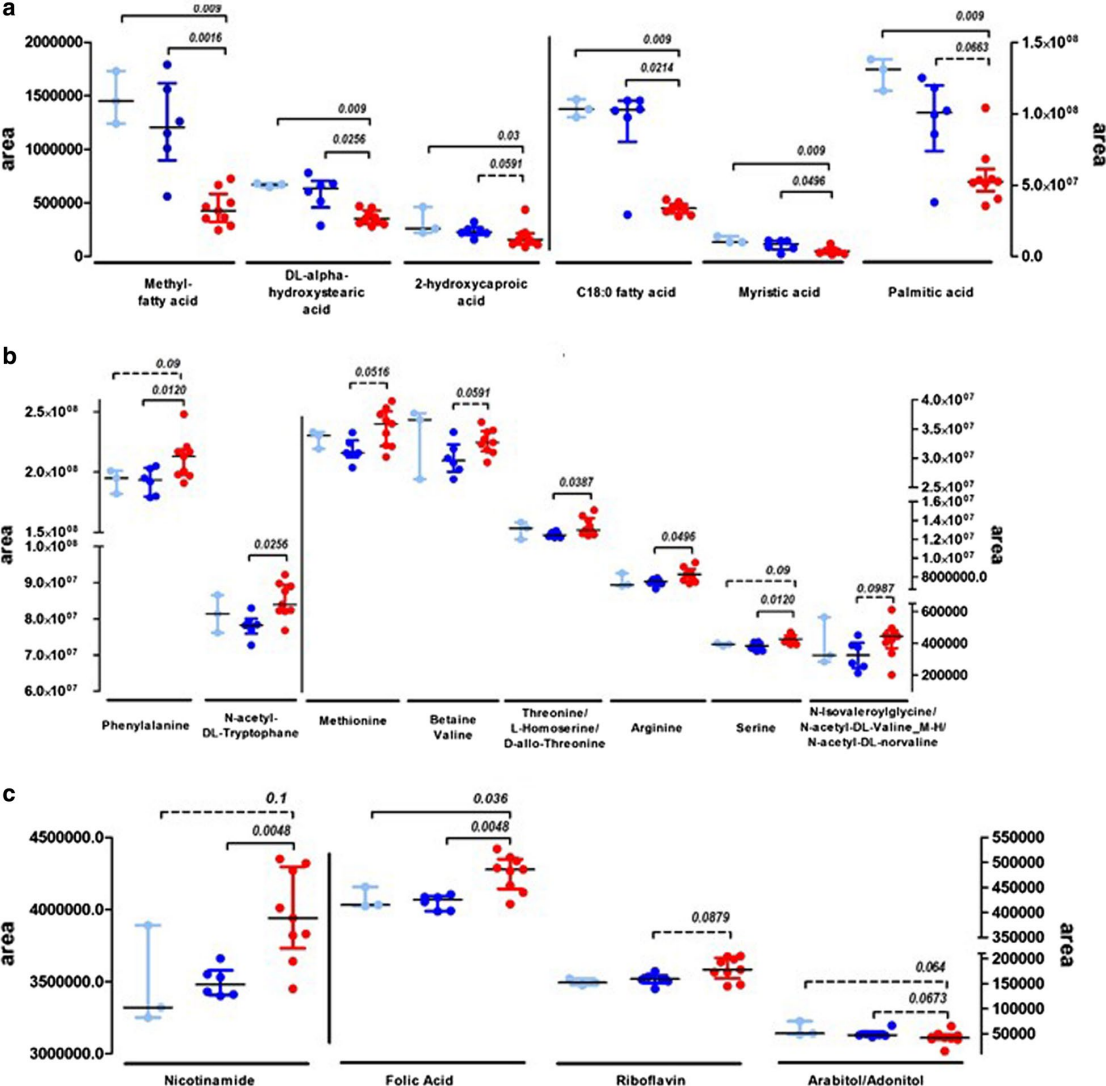
Plasma metabolites in samples from CM-FPIES, IgE-CMA or resolved IgE-CMA patients. **a** Discriminant metabolites between CM-FPIES (red symbols), IgE-CMA (dark blue symbols) and IgE-resolved (light blue symbols). **a** Fatty acids, **b** amino acids and their derivatives, **c** purine metabolites or vitamins. The *p* values of nonparametric Mann–Whitney statistical test are indicated

Non-targeted metabolomics analysis was performed on plasma collected before the OFC from children with CM-FPIES and IgE-CMA, and in 3 children initially recruited for IgE-CMA but who experienced negative OFC (IgE-resolved). Metabolic profiles were obtained using two complementary LC–MS methods and analyzed by univariate analysis. We could then identify metabolites discriminating our different CMA patients (Fig. 8 and Table 3). Some fatty acids significantly discriminated CM-FPIES patients from active and resolved IgE-CMA (Fig. 8a): in CM-FPIES, we observed significantly lower concentrations of alpha-hydrostearic acid, 2-hydroxycaproic acid, myristic acid, palmitic acid, and other unidentified methyl and saturated fatty acids. Conversely, higher levels of some amino acids and their derivatives, purine metabolites or vitamins were observed in CM-FPIES patients when compared to IgE-CMA patients, but less clearly compared to IgE-resolved ones (Fig. 8b, c).

**Table 3:** Plasma metabolites discriminating CM-FPIES versus IgE-CMA patients

Metabolites	*p* value (FPIES vs IgE)*	Formula	Mass	Chemical class	Kegg number	RT ZIC-pHILIC (min)	RT C18 (min)	Identification level
Arginine	*0.0496*	C6H14N4O2	174.1117	Amino acids, peptides and analogues	C00062	–	0.78	1
Serine	*0.0156*	C3H7NO3	105.0426		C00065	–	0.83	1
N-Acetyl-DL-tryptophan	*0.0256*	C13H14N2O3	246.1004		NA	–	6.20	1
l-Cysteine S-sulphate	*0.0496*	C3H7NO5S2	200.9766		C05824	10.14	–	1
Alpha-d-Aminoadipic acid	*0.0496*	C6H11NO4	161.0688		C00956	9.21	–	1
Threonine/l-homoserine/d-allo-threonine	*0.0386*	C4H9NO3	119.0582		C00188/C00263/C05519	7.37	–	3
Methionine	0.0516	C5H11NO2S	149.0511		C00073	–	1.05	1
Phenylalanine	0.0516	C9H11NO2	165.0790		C00079	–	2.09	1
N-Isovaleroylglycine/N-acetyl-DL-valine/N-acetyl-DL-norvaline	0.0987	C7H13NO3	159.0895			2.19	–	3
Betaine/valine	0.0591	C5H11NO2	117.0790		C00719/C00183	5.19	–	1
Nicotinamide	*0.0048*	C6H6N2O	122.0480	Aromatic heteromonocyclic compounds	C00153	–	1.00	1
Theobromine	*0.0360*	C7H8N4O2	180.0647		C07480	–	2.38	1
Folic acid	*0.0048*	C19H19N7O6	441.1397		C00504	–	4.64	1
Uric acid	0.0673	C5H4N4O3	168.0283		C00366	–	0.94	1
Hypoxanthine	0.0663	C5H4N4O	136.0385		C00262	–	0.99	1
Riboflavin	0.0872	C17H20N4O6	376.1383		C00255	–	5.18	1
3-Hydroxydecanoic acid/10-hydroxydecanoic acid	*0.0447*	C10H20O3	188.1412	Organic acids and derivatives	C02774	1.42	–	3
2-Hydroxycaproic acid	0.0591	C6H12O3	132.0786		NA	2.11	–	1
d-Galacturonic acid	*0.0390*	C6H10O7	194.0426	Carbohydrates and carbohydrate conjugates	C08348	9.37	–	1
l-Arabitol/adonitol/d-arabitol	0.0673	C5H12O5	152.0685		C00532/C00474/C01904	6.40	–	1
Myristic acid	*0.0496*	C14H28O2	228.2089	Lipids	C06424	1.36	–	1
DL-Alpha-hydroxystearic acid	*0.0256*	C18H36O3	300.2664		NA	1.34	–	1
Methyl fatty acid	*0.0016*	C15H30O2	242.2246		NA	1.35	–	3
C18:0-FA	*0.0214*	C18H36O2	284.2715		NA	1.34	–	3
Palmitic acid	0.0663	C16H32O2	256.2402		C00249	1.35	–	1

p < 0.05 values are shown in italic

The discriminant metabolites were identified using nonparametric Mann–Whitney test. The metabolites were considered as discriminant when *p* value < 0.1

## Discussion

In this study, we show that both humoral and cellular responses to relevant CM components are poor in children with CM-FPIES. In these patients, the levels of total and specific Ig differed from those of both milk-tolerant peanut allergic children and children with IgE-CMA who were avoiding milk. Thus, these differences cannot be related to CM consumption or avoidance. Moreover, we found no specific IgE against polypeptides derived from gastric and gastroduodenal digestion of CM proteins. This finding does not support the hypothesis that patients with FPIES have specific IgE against neo-epitopes generated during gastroduodenal digestion. Our findings confirm previous data showing a poor humoral response in FPIES, and extend these results to other CM components and their digestion products, and to all Ig types [10, 33, 34]. Our findings also further support that this low level of humoral response is a feature of the disease itself, and is not dependant on milk avoidance.

Interestingly, IgG4 binding to gastroduodenal digestion products of CM proteins differed between children with CM-FPIES and IgE-CMA. We can therefore speculate that IgG4 epitopes might differ between CM-FPIES and IgE-CMA, being more resistant to digestion in IgE-CMA.

Our in-depth cellular analyses showed that in children with CM-FPIES, isolated PBMC were unable to secrete significant amounts of Th cytokines upon CM stimulation, and that Th memory cell proliferation was not detectable after stimulation with CM. Conversely, in children with IgE-CMA, significant secretions of IL-5, IL-13, IFN$\upgamma$ and to a lesser extent of IL-17 were found, and proliferation of Th cells was observed. Our results are not in line with those of Morita et al. showing that reactivated PBMC from patients with non-IgE CMA produce high levels of Th2 and Th1 cytokines [24]. However, their patients were younger and had heterogeneous phenotypes, with 52% having FPIES, and 41.5% and 6.5% presenting food protein-induced proctocolitis or enteropathy syndrome, respectively. In addition, the authors did not report any CM-induced cytokine secretion in their IgE-CMA patients. Caubet et al. found significant secretion of IL-5, IL-13 and IFN$\upgamma$ after casein stimulation in children with both CM-FPIES and IgE-CMA, and low secretion of IL-10. However, Th2 cytokine secretion was comparable between children with CM-FPIES and control subjects tolerating CM [10]. Differences in the age or phenotype of the patients, or the fact that PBMC were obtained after the OFC in the study by Caubet et al., may explain the differences with our results. In addition, our patients with CM-FPIES reacted during the OFC without having any detectable specific memory Th cells before the OFC. This suggests that these cells may not be involved in clinical reactivity, supporting that subjects with active FPIES do not have elevated numbers of food responsive T-cells compared to healthy control subjects before or after the OFC [9]. In line with previous studies, we found significant allergen-specific secretion of the pro-inflammatory cytokines IL-6 and TNF-$\upalpha$ in children with CM-FPIES, albeit lower than that observed in IgE-CMA patients [10].

The presence of increased numbers of eosinophils and other plasma cells in the lamina propria of patients with FPIES supports the presence of neutrophils, eosinophils and other mononuclear cells found in faecal mucus of positive challenge cases [35, 36]. Faecal extracts obtained after milk challenge have shown a high concentration of eosinophil-derived neurotoxin that might be due to a reduction in epithelial barrier function and increased eosinophil degranulation [37]. All these observations may question the role of the adaptive immune response in FPIES, in line with a recent hypothesis suggesting that FPIES resembles the innate response to bacterial infection and may result from abnormalities in the innate immune system, which misrecognizes specific foods [38] and/or which may over-respond to a dysbiotic microbiota, a question that has not been assessed to date. However, our preliminary cytometry analysis of biopsy samples found activated Th1, Th2 and Th17 cells in the mucosa of active FPIES patient, suggesting involvement of the local adaptive immune system in the pathophysiology of FPIES. This may largely explain the specificity of the clinical response, which is triggered only by specific foods, but also the occurrence of symptoms restricted to the gastrointestinal tract and their delayed onset. Cellular analysis of biopsy samples also revealed the presence of other cell types in the mucosa of patients with active FPIES, including ILC. These mucosal ILC mainly have a mixed ILC2/ILC1 phenotype, probably induced by the local inflammatory microenvironment, reflecting ILC plasticity in tissue [39]. These results are in line with the recently reported systemic antigen-specific activation of innate cells associated with positive food challenge [9], but ILC were not assessed. In this latter study, a systemic antigen-specific activation of innate cells, involving monocytes, neutrophils, eosinophils, and NK cells, was associated with a positive food challenge. In this study and ours, the small sample sizes preclude definitive conclusions, and the results need to be confirmed.

No predictive biomarkers are available to date to improve the diagnosis of active FPIES, and to avoid a stressful OFC. A trend to increased serum glutamic oxaloacetic transaminase and lactate dehydrogenase levels in resting conditions was noted, compared with normal ranges in most of the patients who further experienced a positive OFC or acute accidental episodes, which may suggest some intestinal cellular damage [38]. However, the predictive value of this increase has to be validated. Our non-targeted metabolomics approaches performed using plasma collected before the OFC show that CM-FPIES patients are characterized by a specific metabolic profile, for example with lower concentrations of some fatty acids in plasma. Interestingly, besides the role of fatty acids in membrane biosynthesis and energy supply, interference with their endogenous synthesis has profound effects on the metabolic programming of T cells and finally on the development of Th, notably Th17, and Treg cells. In fact, the glycolytic-lipogenic axis is crucial for Th17 development, but not for that of Treg cells, which require exogenous fatty acids [40–42]. Moreover, protein acetylation, N-myristoylation and palmitoylation, which depends on the corresponding availability of fatty acids, are crucial for many T cell functions, as for example palmitoylation of Ras [43] or N-myristoylation LcK [44] that were shown to be necessary for T-cell activation after TCR engagement. This, like altered metabolism of amino acids, purine compounds or vitamins, clearly warrants further investigation with a larger well-characterized cohort.

Our study has several limitations. First, the sample size is small and we couldn’t include healthy non-atopic age-matched control children due to ethical reasons. However, our analyses were robust and included all major CM constituents in a well-phenotyped population. In addition, most of our children with CM-FPIES tolerated small amounts of CM, and thus may be outgrowing the disease. Their immunologic response may therefore differ from that of children with active disease. It is not recommended to perform an OFC for the diagnosis of FPIES, so further analysis should be performed shortly after an acute episode in patients with active CM-FPIES. Finally, children with IgE-CMA tolerated baked milk on the day of their OFC, thus their allergy was probably less severe than those who cannot tolerate either raw or baked milk. These children may have higher levels of Ig, particularly IgG4 subtypes, than those who are allergic to both forms of CM.

## Conclusions

Systemic antigen-specific T-cell and humoral responses were not found in our CM-FPIES patients,  which cannot be attributed to a lower exposure to cow’s milk. However, very preliminary data obtained on intestinal biopsies from one active *versus* one resolved FPIES patients evidenced T-cell infiltrate in active patient, suggesting that adaptive immunity has a role in FPIES pathophysiology, potentially restricted to the intestinal mucosa. Our preliminary data also suggest that new studies analyzing innate cells, including ILC, may help to delineate the pathophysiology of FPIES. Finally, metabolomics approaches could be useful to identify biomarkers for FPIES, highlighting altered metabolic pathways in biofluids.

## Additional files

**Additional file 1.** Cytokine secretion after mitogen reactivation of PBMC

**Additional file 2.** Analysis of memory cells within PBMC before and after reactivation using PHA mitogen

## Abbreviations

CMA: Cow’s milk allergy
FPIES: Food Protein-Induced Enterocolitis Syndrome
PNA: peanut allergy
BLG: beta-lactoglobulin
α-lact: alpha-lactalbumin
LF: lactoferrin
cas: whole caseins
αs1-cas: alpha-s1 casein
αs2-cas: alpha-s2 casein
β-cas: beta-casein
κ-cas: kappa-casein
