# Supplementary material for: Deep analysis of immune response and metabolic signature in children with food protein induced enterocolitis to cow’s milk
Source: Clin Transl Allergy. 2018 Sep 28;8:38. doi: 10.1186/s13601-018-0224-9 (PMC6161449; doi:10.1186/s13601-018-0224-9)

Additionnal file 1 – Cytokine secretion after mitogen reactivation of PBMC

Cytokines secreted after nonspecific reactivation of PBMC from IgE-CMA (blue) or FPIES-CMA (red) patients. PBMC were stimulated for 6 days with 1 µg/ml of PHA (**A**) or LPS (**B**) and cytokines assayed in supernatants using Luminex® xMap® technology and kits and apparatus from Bio-rad. Results are expressed as medians obtained for the PBMC from IgE-CMA or FPIES-CMA patients. Statistics: * indicated a significant difference between non-stimulated (media alone) and stimulated PBMC within the same group of patient (p<0.05 using Wilcoxon sign rank test). Trend between IgE-CMA and FPIES-CMA patients are also indicated (Mann Whitney test).

A

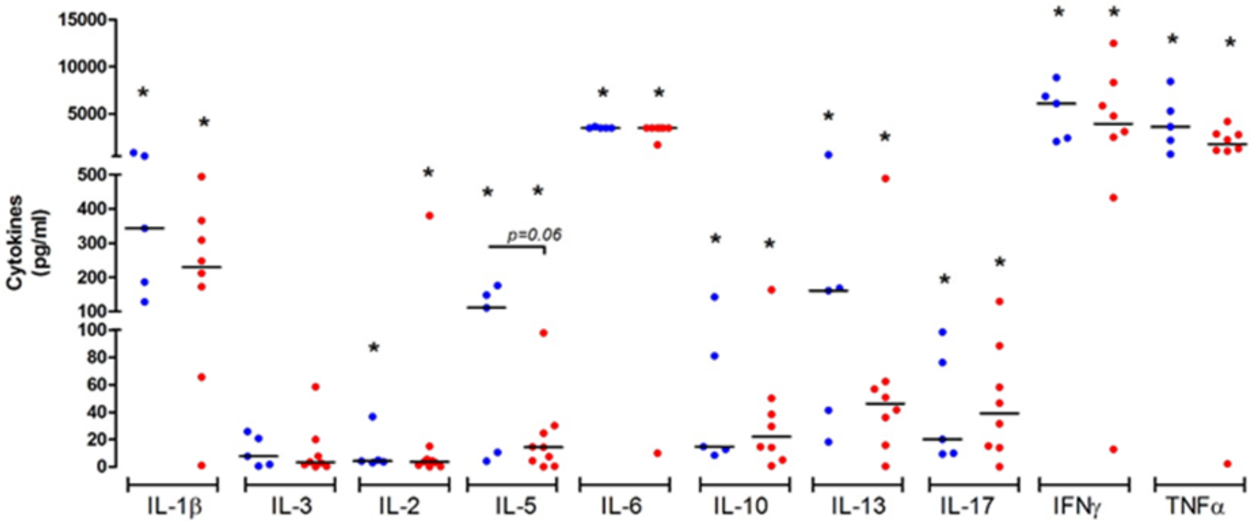

B

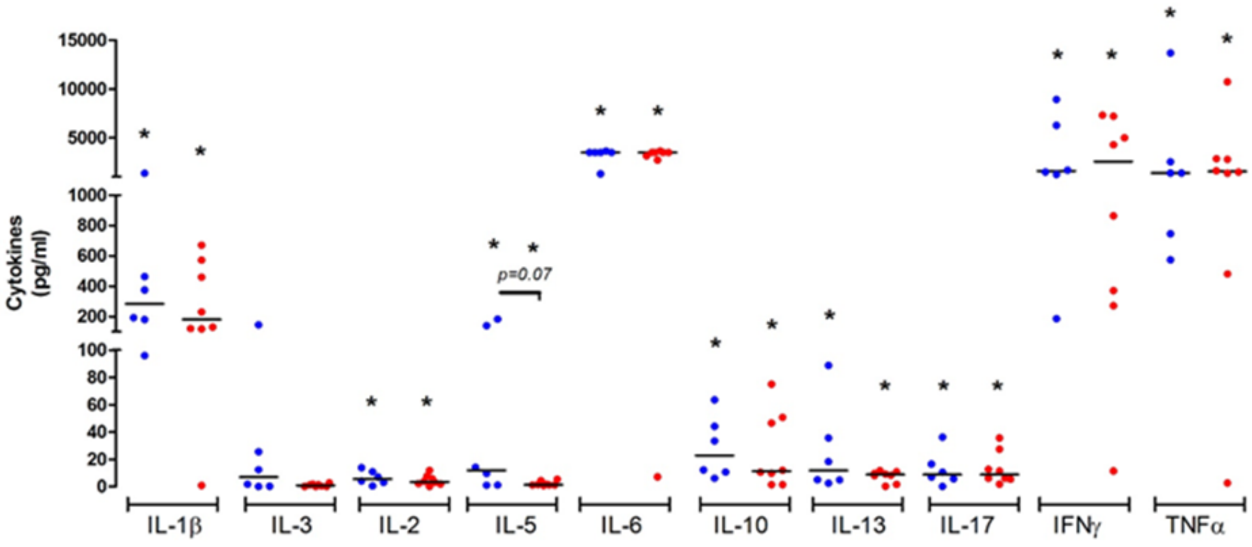

Supplement: Supplementary file 1 — Additional file 1. Cytokine secretion after mitogen reactivation of PBMC [file 13601_2018_224_MOESM1_ESM.pdf]
