# Supplementary material for: Deep analysis of immune response and metabolic signature in children with food protein induced enterocolitis to cow’s milk
Source: Clin Transl Allergy. 2018 Sep 28;8:38. doi: 10.1186/s13601-018-0224-9 (PMC6161449; doi:10.1186/s13601-018-0224-9)
Supplement: Supplementary file 2 — Additional file 2. Analysis of memory cells within PBMC before and after reactivation using PHA mitogen [file 13601_2018_224_MOESM2_ESM.pdf]

Additionnal file 2 : Analysis of memory cells within PBMC before and after reactivation using PHA mitogen

Memory Th1, Th2, Th17 and Treg cells in non-stimulated (**A**) and PHA-activated (**B**) PBMC. *Analysis of T helper memory cells*: after 6 days of culture, PBMC were collected and labelled extracellularly with anti-human CD4, CD25, CD45RA, CCR6, CXCR3 and CCR4. Gating strategies were performed as described in [1], to analyse memory Th1, Th2, Th17 and Th1* cells. Within $CD4^+$ single cells, $CD25^-CD45RA^-$ memory cells were selected, and further analysed for CCR6, CXCR3 and CCR4 expression. Th1 memory cells (mTh1, ■) correspond to $CXCR3^+CCR4^-$ cells within $CCR6^-$ population, Th2 memory cells (mTh2, ■) correspond to $CXCR3^-CCR4^+$ cells within $CCR6^-$ population, Th17 memory cells (mTh17, ■) to $CXCR3^-CCR4^+$ cells within $CCR6^+$ population and non-conventional Th1 memory cells (mTh1*) to $CXCR3^+CCR4^-$ cells within $CCR6^+$. This latter population was not numerous enough to be analysed (not shown). Results are expressed as percentage of corresponding cells within the selected $CD25^-CD45RA^-$ population of $CD4^+$ single cells. *Analysis of Treg cells:* after 6 days of culture, PBMC were collected and labelled extracellularly with anti-human CD3, CD4, CD25 and intracellularly with Foxp3. Treg cells (☐) were defined as $CD25^+Foxp3^+$ population within CD3+CD4+ single cells.

* indicates a significant difference between IgE-CMA and FPIES-CMA patients ($p<0.05$, Mann Whitney test).

A

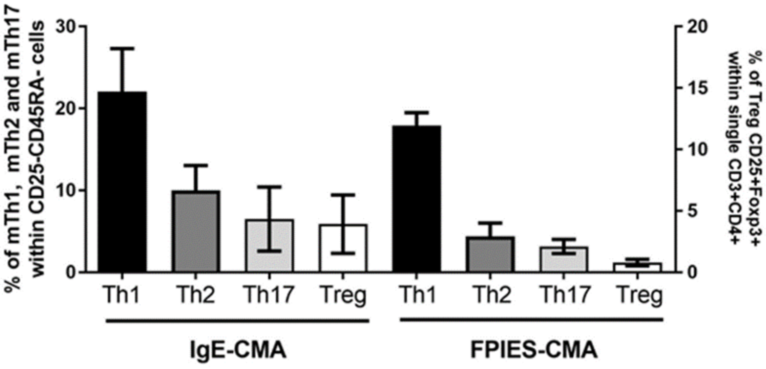

B

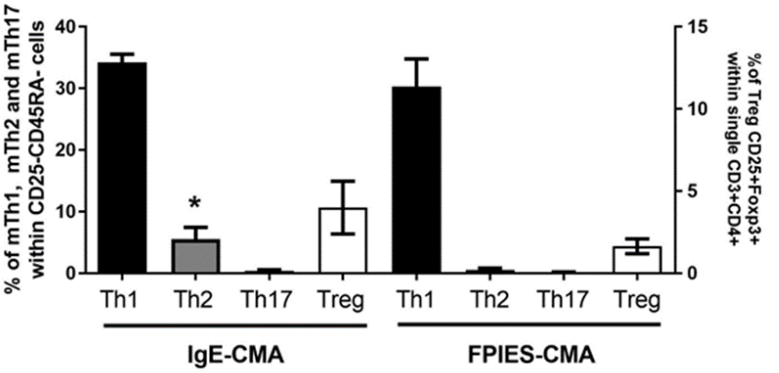

1. Becattini S, Latorre D, Mele F, Foglierini M, De GC, Cassotta A, Fernandez B, Kelderman S, Schumacher TN, Corti D *et al*: T cell immunity. Functional heterogeneity of human memory CD4(+) T cell clones primed by pathogens or vaccines. Science. 2015; 347(6220):400-406.
